# Rebooting the Adaptive Immune Response in Immunotherapy‐Resistant Lung Adenocarcinoma Using a Supramolecular Albumin

**DOI:** 10.1002/smll.202404892

**Published:** 2024-10-21

**Authors:** Fanni Li, Jingmei Wang, Tianya Liu, Wenguang Yang, Yong Li, Qi Sun, Jin Yan, Wangxiao He

**Affiliations:** ^1^ Department of Medical Oncology and Department of Talent Highland The First Affiliated Hospital of Xi'an Jiaotong University Xi'an 710061 P. R. China; ^2^ Institute for Stem Cell & Regenerative Medicine The Second Affiliated Hospital of Xi'an Jiaotong University Xi'an 710004 China; ^3^ Department of infectious Diseases and Department of Tumor and Immunology in precision medical institute The Second Affiliated Hospital of Xi'an Jiaotong University Xi'an 710004 P. R. China; ^4^ National & Local Joint Engineering Research Center of Biodiagnosis and Biotherapy The Second Affiliated Hospital of Xi'an Jiaotong University Xi'an 710004 P. R. China; ^5^ Department of general surgery The First Affiliated Hospital of Xi'an Jiaotong University Xi'an 710061 P. R. China

**Keywords:** Cancer therapy, immunotherapy, LUAD, Resistance, Wnt inhibitor

## Abstract

Despite the availability of immune checkpoint inhibitors (ICBs) significantly prolonging the life expectancy of some lung adenocarcinoma (LUAD) patients, their implementation and long‐term effectiveness are hampered by the growing issue of acquired resistance. Herein, the bioinformatics analysis of immunotherapy‐resistant LUAD patients and the system analysis of Anti‐PD1‐resistant mice models once again validate that the resistance‐associated Wnt/β‐catenin pathway offers a promising avenue for ICB sensitization. Consequently, a mild and convenient self‐assembly between albumin and carnosic acid (CA), a Wnt inhibitor is employed, to develop a supramolecular albumin known as ABCA, serving as a reactivator for ICB. As anticipated, ABCA effectively suppress the Wnt/β‐catenin cascade in vitro and leads to significant inhibition of cell proliferation while promoting apoptosis. Most notably, ABCA restores the anticancer efficacy of Anti‐PD1 in immunotherapy‐resistant LUAD orthotopic allografting mice models by reinvigorating the adaptive immune response mediated by T lymphocytes. Furthermore, ABCA exhibits minimal adverse effects during treatment and high‐dose toxicity tests, underscoring its excellent potential for clinical translation. Collectively, the present work possesses the potential to provide innovative perspectives on the advancement of optimized immunotherapies targeting drug resistance, while also presenting a promising avenue for translating Wnt inhibitors into immunotherapeutic drugs for their clinical application.

## Graphical abstract

Immune checkpoint inhibitors have improved survival for some lung adenocarcinoma (LUAD) patients, but acquired drug resistance limits their effectiveness. To address this, a supramolecular albumin (ABCA) by combining albumin with the Wnt inhibitor carnosic acid (CA) is developed. ABCA reactivates the anticancer effects of anti‐PD1 therapy in an immunotherapy‐resistant LUAD mouse model by reactivating the adaptive immune response.

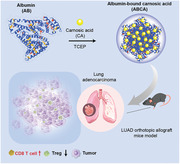

## 1 Introduction

The highly malignant lung adenocarcinoma (LUAD), which constitutes more than two‐third of non‐small cell lung cancer cases as the most prevalent histological subtype, encounters a therapeutic dilemma characterized by low 5‐year survival rates following surgery and limited efficacy of chemotherapy in advanced stages subsequent to targeted therapy failure.^[^ ^1^, ^2^ ^]^ The emergence of immunotherapy, particularly the PD‐1/PD‐L1 immune checkpoint inhibitors (ICBs), has revolutionized the status and significantly prolonged the survival of patients with advanced lung adenocarcinoma (LUAD).^[^ ^1^, ^3^, ^4^, ^5^ ^]^ However, the acquired resistance posed a grave threat to the lives of even those patients who benefited from ICB therapy, resulting in dismal 5‐year survival rates of less than 10%.^[^ ^6^, ^7^, ^8^ ^]^ The accumulating evidence suggests that altered immune microenvironments play a crucial role in acquired immune resistance.^[^ ^9^, ^10^, ^11^ ^]^ In these instances, on one hand, the eradication or suppression signaling of immune cell effectors and oncogenes that are conducive to immunotherapy response in mouse models of PD‐L1 inhibitor resistance and patients with advanced tumor recurrence disrupts normal PD‐L1 expression and facilitates resistance generation;^[^ ^12^, ^13^, ^14^ ^]^ on the other hand, it has been observed in ICB‐resistant tumors that multiple effector T cells such as cytotoxic T lymphocytes and neoantigen‐specific T cells fail to activate, thereby contributing to the incapacity for sustained immune response to ICB immunotherapy.^[^ ^15^, ^16^ ^]^

To surmount this obstacle, a myriad of synergistic ICB therapies have emerged, encompassing but not limited to the combination of Anti‐PD1/PD‐L1 with radiotherapy, chemotherapy, tumor vaccines, or oncolytic viruses, as well as multiple immune checkpoint co‐inhibition.^[^ ^17^, ^18^ ^]^ The aforementioned approaches, while benefiting to a certain extent from the enhanced impact of tumor cell eradication, are constrained by the limited duration and scope of immune modulation within the tumor microenvironment; furthermore, they are plagued by detrimental effects on surrounding normal cells and aberrant inflammatory responses.^[^ ^19^ ^]^ Therefore, precision medicine has been garnering increasing attention due to its focus on targeting specific abnormal proteins in order to rectify immune microenvironments that are not conducive to immune activation. By this way, the pivotal role of β‐catenin in the Wnt pathway has attracted considerable interest as a promising target, owing to its crucial involvement in tumor immune evasion, particularly in conferring resistance to ICBs.^[^ ^20^, ^21^, ^22^ ^]^ It has been demonstrated that β‐catenin hyperactivation is intimately associated with the expression of immunosuppressive cytokines, and various β‐catenin‐activated tumors exhibit reduced infiltration of dendritic cells and T cells, promoting the formation of a tumor‐immune resistant microenvironment.^[^ ^13^, ^23^, ^24^, ^25^ ^]^ To overcome this challenge, emerging reports, including our previous findings, have demonstrated the promising potential of combining Wnt/β‐catenin signaling inhibitors with immune checkpoint blockers (ICBs) in inducing sensitization to immunotherapy in various mouse models of tumor types.^[^ ^26^, ^27^, ^28^, ^29^ ^]^ Nevertheless, the utilization of these immunotherapy sensitizers necessitates meticulous consideration of timing and dose ratios across various administrations, which is constrained by systemic adverse effects associated with the presence of immune‐related targets throughout the body. Furthermore, inadequate tumor accumulation and potential overlapping side effects pose significant challenges.^[^ ^30^ ^]^ As a result, despite Wnt/β‐catenin targeting inhibitors being potential ameliorators for ICB resistance, none of the feasible strategies specifically circumventing or reversing acquired ICB resistance have been approved for clinical application.

To address this challenge, our group has previously reported a small‐molecule compound carnosic acid (CA) as a specific inhibitor of β‐catenin in the Wnt/β‐catenin signaling pathway, which directly binds to and disrupts H1 helices in the armadillo repeat structural domain structural domain of β‐catenin, thereby inducing β‐catenin oligomerisation and degradation.^[^ ^31^ ^]^ To enhance the tumor specificity of therapeutic agents, a multitude of highly efficient nanomedicines have been developed to optimize molecule enrichment within the tumor and ensure their safe circulation. Regrettably, these challenges persist despite advancements in nanomedicine constructions, including issues with inadequate nanoparticle splitting, insufficient compound loading, and complex/costly assembly processes.^[^ ^32^, ^33^ ^]^ Consequently, we herein employed a mild and convenient self‐assembly between albumin and CA, to develop an albumin‐bound carnosic acid known as ABCA, which serves as a reactivator for ICB. As anticipated, ABCA effectively suppressed the Wnt/β‐catenin cascade in vitro and leads to significant inhibition of cell proliferation while promoting apoptosis. Most notably, ABCA restored the anticancer efficacy of Anti‐PD1 in immunotherapy‐resistant LUAD orthotopic allografting mice models by reinvigorating the adaptive immune response mediated by T lymphocytes. Furthermore, ABCA exhibited minimal adverse effects during treatment and high‐dose toxicity tests, underscoring its excellent potential for clinical translation. Collectively, the present work possesses the potential to provide innovative perspectives on the advancement of optimized immunotherapies targeting drug resistance, while also presenting a promising avenue for translating Wnt inhibitors into immunotherapeutic drugs for their clinical application.

## 2 Results

### 2.1 The Resistance to Anti‐PD1 Immunotherapy in NSCLC is Associated with the Activation of the Wnt/β‐Catenin Pathway

**Figure 1:**
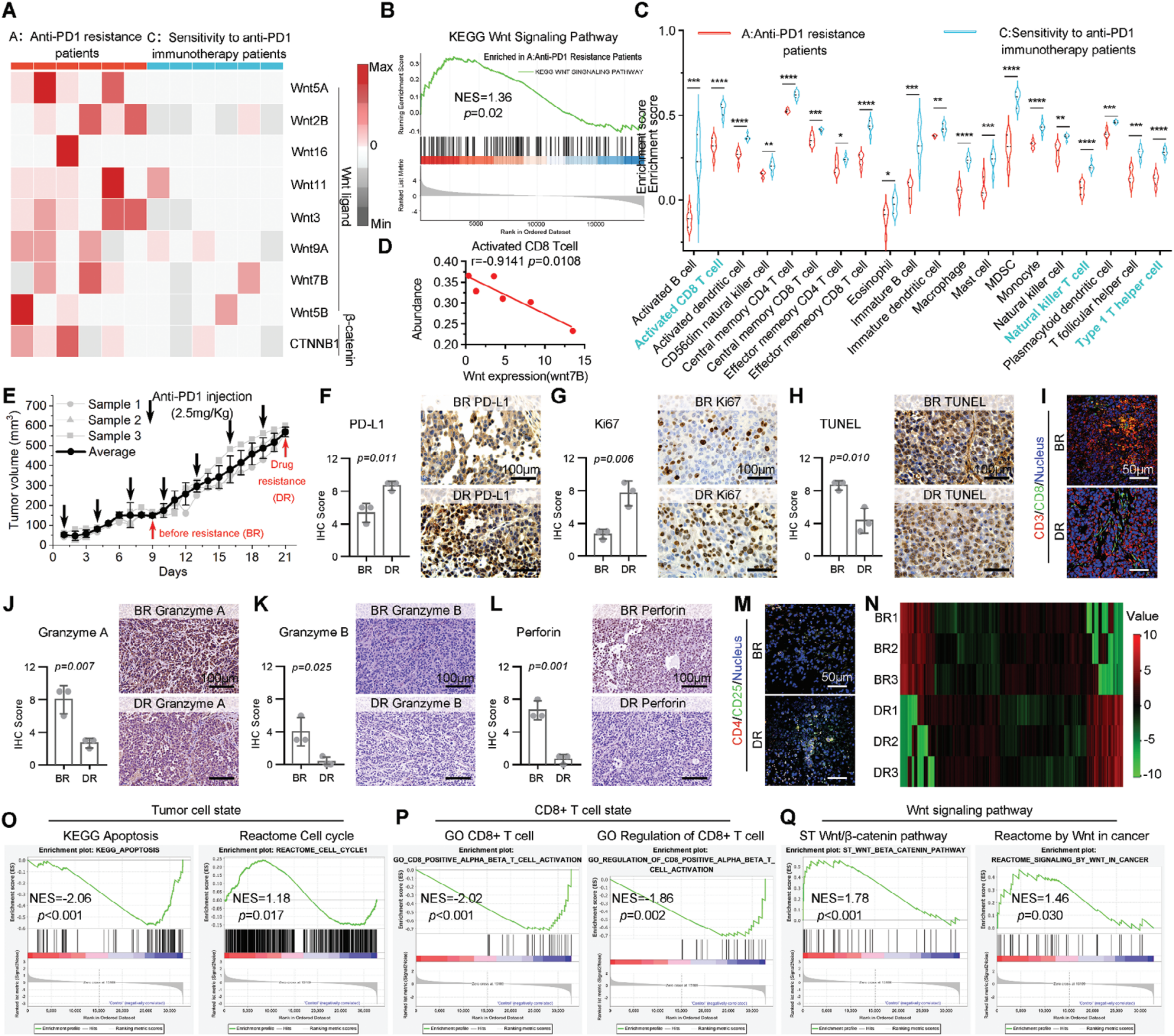
The mechanism of resistance to PD1 immunotherapy associated with Wnt/β‐catenin signaling pathway in NSCLC patients and mice lung adenocarcinoma subcutaneous tumor models. A) Heatmap for Wnt/β‐catenin signaling gene expression in two groups NSCLC patients (*N* = 12, GSE181820) (A: resistance to anti‐PD1 immunotherapy; C: sensitivity to anti‐PD1 immunotherapy). B) GSEA result for the Wnt signaling pathway enriched in A group resistance to anti‐PD1 immunotherapy. C) The comparison of 20 immune cells’ infiltration levels in two groups (A: resistance to anti‐PD1 immunotherapy; C: sensitivity to anti‐PD1 immunotherapy). P values were determined by student's *t‐*test: *, *p *< 0.05; **, *p *< 0.01; ***, *p *< 0.001; ****, *p *< 0.0001. D) Pearson relation analysis showed that Wnt expression (WNT 7B) negative correlated with activated CD8 T cells. E) Tumor growth curve of anti‐PD1 resistance mice lung adenocarcinoma subcutaneous tumors model. F) IHC of PD‐L1 expression and score of mice tumor tissue both before resistance (BR) and drug resistance (DR) (Scale bar: 100 µm). G) IHC of Ki67 expression and score of mice tumor tissue both BR and DR (Scale bar: 100 µm). H) TUNEL staining and IHC score of mice tumor tissue both BR and DR. (Scale bar: 100 µm). I) Immunofluorescence (IF) images of CD3/CD8/nucleus of mice tumor tissue both BR and DR (Scale bar: 50 µm). J) IHC of Granzyme A expression and score of mice tumor tissue both BR and DR. (Scale bar: 100 µm). K) IHC of Granzyme B expression and score of mice tumor tissue both BR and DR. (Scale bar: 100 µm). L) IHC of perforin expression and score of mice tumor tissue both BR) and DR. (Scale bar: 100 µm). M) IF of CD4/CD25/nucleus of mice tumor tissue both BR and DR. (Scale bar: 50 µm). N) Hierarchical clustering of differentially expressed genes between two groups (BR and DR), each row represents one sample, and columns represent genes. O) GSEA result for the cell apoptosis and cell cycle in the DR group. P) GSEA result for the CD8 T cells and regulation CD8 T cells in the DR group. Q) GSEA result for the wnt signaling pathway in the DR group.

The acquired resistance observed in immune checkpoint therapy typically manifests as a phenotype characterized by insufficient immune infiltration.^[^ ^9^ ^]^ To investigate the oncogenic signaling pathways associated with acquired resistance to immune checkpoint inhibitors, we categorized LUAD samples treated with Anti‐PD1 therapy from the GSE database into two distinct cohorts: those demonstrating resistance to Anti‐PD1 therapy and those exhibiting sensitivity to Anti‐PD1 therapy. In the comparison of gene expression profiling, a significant disparity in the expression of Wnt ligand was observed between the two groups. Further, samples demonstrating resistance to Anti‐PD1 therapy exhibited elevated expression levels of β‐catenin (CTNNB1), a pivotal key component involved in the activation of Wnt signaling cascades (**Figure** **1**A). The gene set enrichment analysis (GSEA) also revealed significant enrichment of the Wnt signaling pathway in the group exhibiting resistance to Anti‐PD1 treatment (Figure 1B). The anti‐PD1‐resistant group exhibited significantly reduced infiltration of immune cells, including activated CD8 T cells, natural killer T cells, and Type 1 T helper cells, in comparison to the anti‐PD1‐sensitive group (Figure 1C,D). The collective findings provide support for the acquired resistance concept that an overactive Wnt/β‐catenin signaling pathway is associated with inadequate infiltration and activation of tumor immune cells following Anti‐PD1 therapy.

To validate the conclusions derived from the clinical data, we utilized a PD‐1 resistance animal model and investigated the potential role of aberrant β‐catenin activation in facilitating tumor immunosuppression. The LLC Lewis homograft models of murine lung adenocarcinoma, which are resistant to anti‐PD1 immune checkpoint therapy, were administered Anti‐PD1 injections every 3 days at a dosage of 2.5 mg k^−1^g, as depicted in Figure 1E. The initial phase of tumor growth inhibition was observed from Days 1–9, with minimal increase in tumor volume, indicating a weak response to anti‐PD1 therapy. However, starting from Day 10, the tumors exhibited accelerated growth despite the continuous administration of Anti‐PD1, suggesting significant development of drug resistance. The tumor tissues collected on Day 9 and Day 21 of administration were designated as Before Resistance (BR) and Drug Resistance (DR) samples, respectively. Immunohistochemical analysis revealed a significantly elevated level of PD‐L1 in DR compared to BR, indicating that the lack of response to Anti‐PD1 treatment in this model may be attributed to compensatory upregulation of PD‐L1 (Figure 1F). Additionally, this data suggested that T‐cell‐mediated antitumor immune responses may be impaired.^[^ ^34^ ^]^ The ki67 immunohistochemical assay revealed persistent tumor cell proliferation, while TUNEL staining analysis indicated a reduction in apoptosis within the tumor tissues during Anti‐PD1 treatment, suggesting resistance to the current PD1 blockade approach (Figure 1G,H). Additionally, a concurrent reduction in tumor infiltration of cytotoxic T cells was observed alongside the emergence of drug resistance (Figure 1I), as indicated by decreased levels of Granzyme‐A, Granzyme‐B, and Perforin in the DR sample (Figure 1J–L). The immunofluorescence results notably demonstrated a distinct upregulation of regulatory T cells in DR compared to BR, thereby confirming the establishment of an immunosuppressive microenvironment in DR tumor samples. This finding may serve as a crucial factor contributing to the attenuated therapeutic efficacy of Anti‐PD1 (Figure 1M).

Furthermore, transcriptome sequencing (RNA sequencing) was conducted to elucidate the underlying mechanism responsible for the unresponsiveness of the Anti‐PD1‐resistant immune response (Figure 1N). The GSEA analysis once again demonstrated the presence of active tumor proliferation in the DR group, as indicated by the downregulation of apoptosis signaling in tumor cells and the upregulation of the cell cycle pathway (Figure 1O). The insufficiency of immune response was once again confirmed in this group, as evidenced by the suppression of pathways related to cytotoxic T cells (Figure 1P). The DR tumors, in contrast to BR tumors, exhibited a heightened activation of the Wnt/β‐catenin signaling pathway, which aligns with the aforementioned analysis of patient data (Figure 1Q). These findings demonstrate that sustained administration of Anti‐PD1 can lead to acquired resistance through aberrant activation of the Wnt/β‐catenin pathway, resulting in inadequate immune infiltration and an unfavorable immune microenvironment.

### 2.2 The Design and Characterization of a Wnt Inhibitor, Known as ABCA

**Figure 2:**
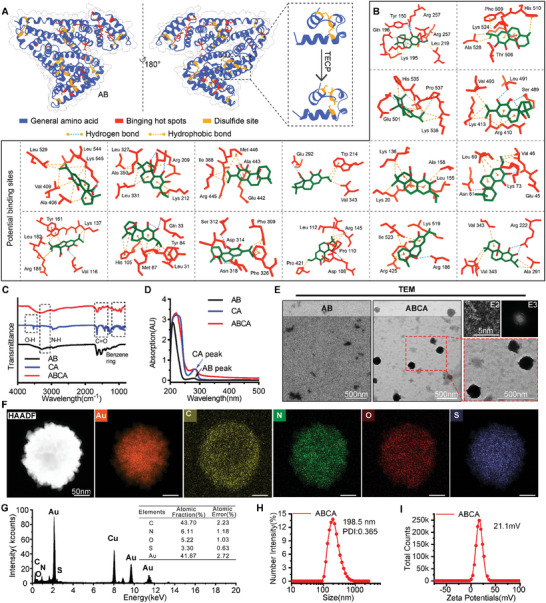
Synthesis and characterization of ABCA. A) The structure of Human Serum Albumin (AB) protein was presented and disulfide bonds of AB were opened by TECP reduced. B) Computer‐aided drug design simulated a series of binding sites for AB and Carnosic acid (CA). C) UV absorption spectra of HSA (black line), CA (blue line), and ABCA (red line). D) FTIR spectra of AB, CA, and ABCA. The characteristic absorption peaks of O─H bonds of CA at 3500 cm^−1^ and N─H bonds of AB at 3200 cm^−1^. E) TEM images of AB and ABCA (the ratio of AB to CA is 5:2) were filmed by transmission electron microscopy. The HRTEM of an individual particle ABCA (E2) and corresponding FFT pattern (E3). F) The element analysis single particle of ABCA (5:2). G) Energy dispersive spectroscopy analysis of ABCA (5:2). H) Hydrodynamic diameter distribution of the ABCA (5:2) measured by dynamic light scattering. I) Zeta potential of ABCA measured at pH 7.4.

After conducting the aforementioned analysis, it appears to be a feasible approach to inhibit the intrinsic active Wnt/β‐catenin pathway in tumors for the purpose of augmenting T‐cell infiltration and overcoming tumor resistance within the context of PD1/PD‐L1 blockade. Therefore, the inhibition of the central component of the Wnt pathway is anticipated to yield improved outcomes. Carnosic acid (CA) has emerged as a notable inhibitor of the central component β‐catenin due to its well‐defined mechanism.^[^ ^35^ ^]^ The utilization of CA is impeded by two primary obstacles: its hydrophobic nature, which complicates its dissolution in the in vivo solution milieu, and its significant toxicity toward normal cells. Overcoming these challenges requires the implementation of improved tumor‐targeting strategies and enhanced biosafety measures for the in vivo application of CA. Motivated by the remarkable biocompatibility and pharmacokinetic characteristics exhibited by albumin (AB),^[^ ^36^, ^37^, ^38^ ^]^ it has been utilized as a carrier for CA to develop a potent Wnt suppressor with enhanced biosafety features. This process involved the utilization of the reducing agent Tris(2‐carboxyethyl) phosphine (TCEP) to disrupt the disulfide bonds of albumin, ultimately resulting in the formation of a self‐assembled nanoparticle (ABCA) through the hydrophobic interaction of CA with AB (**Figure** **2**A,B).

The chemical property of ABCA was validated through Fourier Transform Infrared (FT‐IR) spectroscopy (Figure 2C), revealing the presence of two distinctive absorption peaks corresponding to amide bonds at ≈1500 cm^−1^ (C═O) and 3200 cm^−1^ (N─H) and a hydroxyl group at 3500 cm^−1^(O─H) in the infrared spectra of ABCA (Figure 2C). These findings were further corroborated by the UV–vis spectrum of ABCA, which exhibited characteristic absorption peaks associated with AB (Figure 2D). Moreover, Transmission electron microscope (TEM) images reveal that ABCA exhibits a remarkably uniform size and possesses highly desirable monodisperse characteristics (Figure 2E). The high‐resolution transmission electron microscope (HRTEM) images, combined with energy dispersive spectroscopy analysis, revealed a uniform distribution of elements within the particle, consistent with its components (Figure 2F,G). The ABCA was demonstrated by determining its hydrodynamic diameter as 198.5 nm (Figure 2H) and Zeta potential as 21.1 mV at pH 7.4 (Figure 2I). In addition, ABCA was incubated in a solution containing 20% serum, the particle size of ABCA remained ≈200 nm over a period of 6 days (Figure S1A, Supporting Information). The indicates that ABCA exhibits significant stability. Subsequently, the CA released from ABCA was quantified using high‐performance liquid chromatography. Results indicated that ≈70% of CA was released within 24 h under incubation conditions with DTT, whereas negligible CA release was observed in the absence of DTT (Figure S1B, Supporting Information). The result suggested that its potential for excellent stability and subsequent reductive response release of CA.

### 2.3 The Biosafety Assessment of ABCA In Vivo

**Figure 3:**
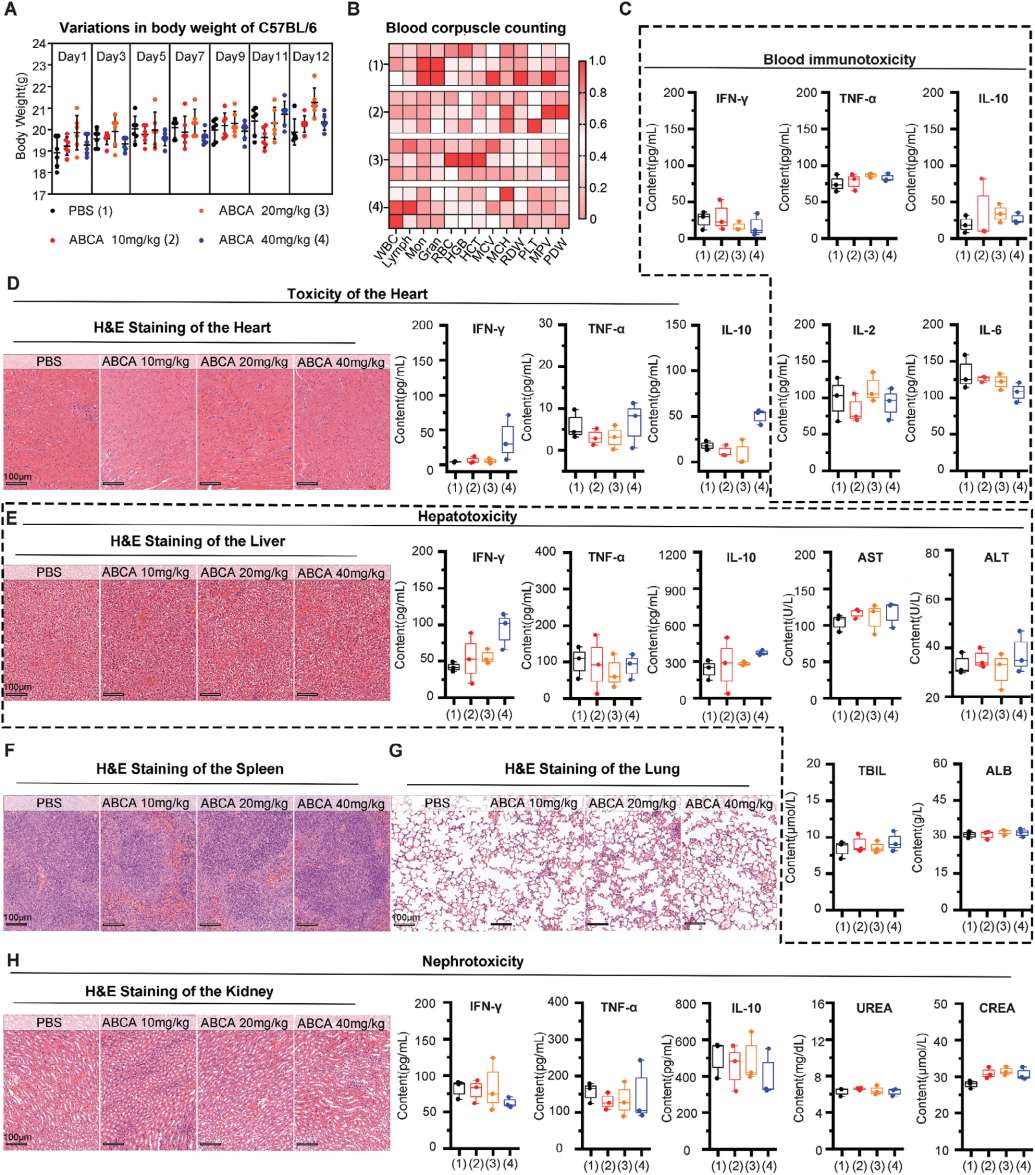
Biosafety assessment of ABCA in mice. A) Variations in body weight of C57BL/6 during the 2 weeks treated with different doses of ABCA administration (*n *= 6 per group). B) Heat map of the relationships between routine blood biochemical indicators and different mouse groups injected with different dosages of ABCA and PBS (*n *= 3 per group). (1): PBS group, (2):1 mg k^−1^g ABCA group, (3):20  mg k^−1^g ABCA group, (4):40 mg k^−1^g ABCA group. C) Blood immunotoxicity measured by the expression of inflammatory factors in serum including IFN‐γ, TNF‐α, IL‐10, IL‐2 and IL‐6 were analyzed by flow cytometry (*n* = 3 per group). D) Heart toxicity assessment of the different dosages of ABCA measured by inflammatory factors (TNF‐α, IL‐10, IFN‐γ *n* = 3 per group) and pathological section of Heat (scale bar: 100 µm). E) Hepatotoxicity assessment of the different doses of ABCA measured by aspartate transaminase (ALT), alanine aminotransferase (AST), total bilirubin(TBIL), albumin (ALB), inflammatory factors (TNF‐α, IL‐10, IFN‐γ *n* = 3 per group) and pathological section of the liver (scale bar: 100 µm). F,G) The representative histological H&E staining images of Spleen F) and Lung G) in C57BL/6 with the indicated treatments (scale bar: 100 µm). H) Nephrotoxicity assessment of the different dosages of ABCA measured by UREA, creatinine (CREA), inflammatory factors (TNF‐α, IL‐10, IFN‐γ *n* = 3 per group), and pathological section of the liver (scale bar: 100 µm).

To assess the in vivo safety profile of ABCA, C57BL/6 mice were randomly divided into four groups and administered either PBS (Control group) or ABCA (*n* = 6 per group) at doses of 10, 20, and 40 mg k^−1^g every other day for a duration of two weeks. The administration of a test dose, ranging from three to over ten times higher than the treatment dose, was observed not to result in any abnormal weight loss in the respective groups of mice, indicating that the mice were not adversely affected by the administration procedure (**Figure** **3**A). The results of the blood routine examination indicated that, in comparison to the control group, the increase in drug concentration had minimal impact on hematological parameters (Figure 3B). Furthermore, there were no significant fluctuations observed in the levels of representative immune factors (TNF‐α, IFN‐γ, IL‐2, IL‐6, and IL‐10) in the blood of mice across various treatment groups (Figure 3C). These results suggest that even when administered at doses several times higher than the standard therapeutic dosage, ABCA does not elicit hematological immune toxicity.

The favorable biosafety profile of ABCA is further demonstrated by its lack of detrimental impact on the overall functioning of organs and the immune system. It is worth noting that Wnt/β‐catenin pathway antagonists and inhibitory small molecules have not yet received clinical approval, primarily due to their associated side effects on normal tissues and organs.^[^ ^39^ ^]^ Encouragingly, the administration of ABCA did not exhibit any observable signs of toxicity‐induced harm to vital organs including the heart, liver, spleen, lung, and kidney, as confirmed by histological examination (H&E staining) and measurements of inflammatory factors (Figure 3D–H). Furthermore, in comparison to the control group, ABCA treatments with various dosages exhibited negligible immunogenic impact on the hepatic and renal function, establishing that ABCA does not impose any detrimental burden on these pivotal metabolic organs (Figure 3E,H). Currently, metabolizable nanotherapeutic agents exhibit reduced toxicity, potentially attributable to their efficient excretion from the body.^[^ ^40^ ^]^ In accordance with our design, ABCA undergoes decomposition into ultra‐small nanoparticles within a reducing in vivo environment, facilitating effective excretion. To validate this hypothesis, we administered ABCA intravenously to C57BL/6 bearing LLC tumor mice, and subsequently detected ^197^Au content in various organs at 0, 4, 6, 18, 24, 48, 120 h, and 7 days post‐injection using ICP‐MS (Figure S2, Supporting Information). The results suggest that ABCA can be metabolized in vivo.

In addition, given the positively charged nature of ABCA, we investigated the potential for haemolysis during in vivo treatment. A haemolysis assay was conducted on ABCA, and the results were compared with a positive control group. The haemolytic activity of ABCA was found to be less than 2% at both the therapeutic dose of 3 mg k^−1^g and the maximum dose of 40 mg k^−1^g (Figure S3, Supporting Information). Consequently, concerns regarding the haemolytic potential of ABCA in vivo were alleviated. The findings suggest a minimal immunogenic response and biological toxicity, indicating that the utilization of ABCA, known for its mild and safe properties, provides an opportunity to evaluate the in vivo immune synergistic effects of CA and explore its potential in combination therapy.

### 2.4 ABCA Achieved Potent Antitumor Effects and Wnt Signaling Cascades Suppression In Vitro

**Figure 4:**
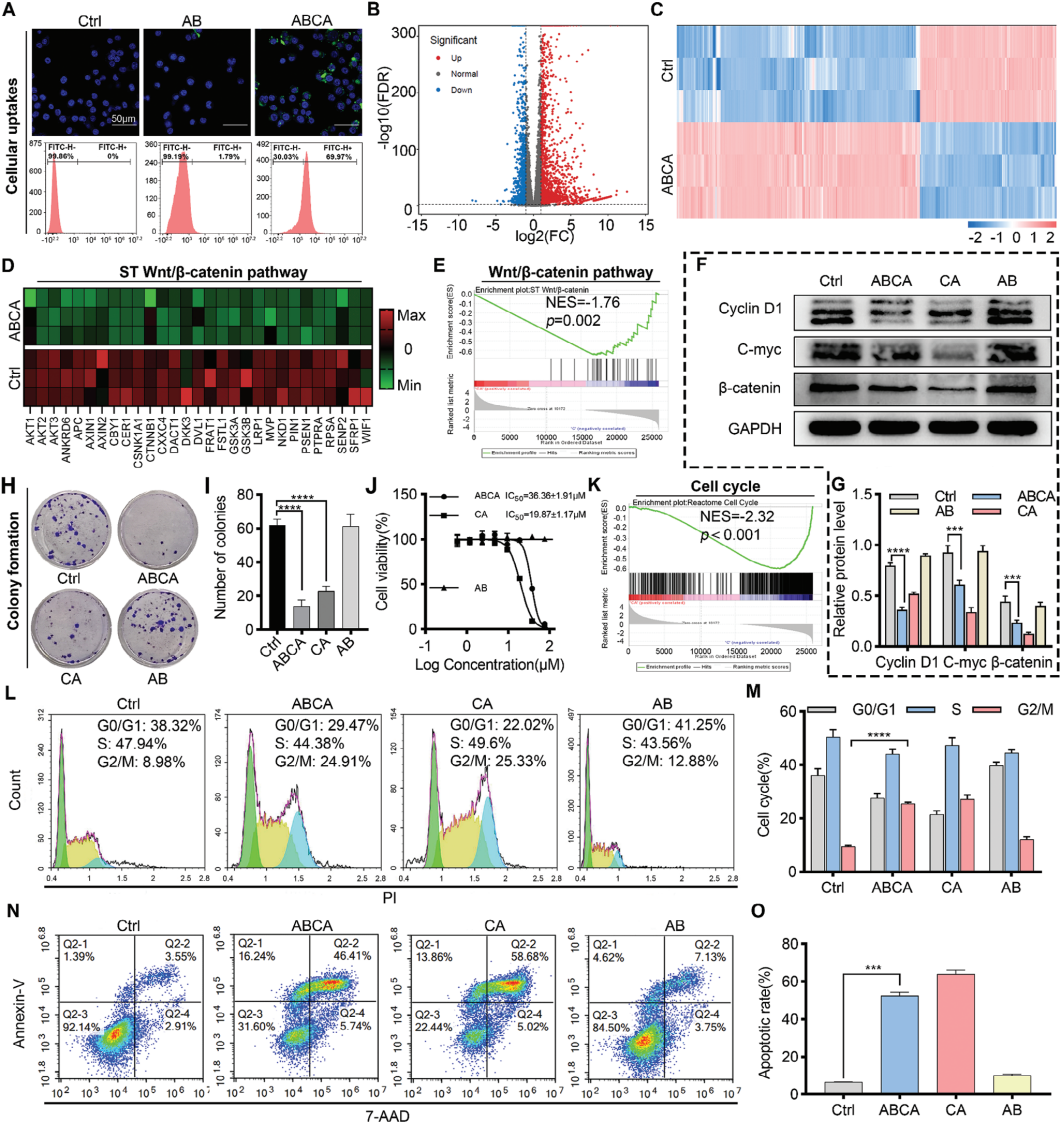
In vitro anticancer efficacy and mechanism of ABCA on lung cancer LLC cells. A) Cellular uptakes of ^FITC^AB and ^FITC^ABCA by LLC cells at 6 h, observed by fluorescence microscope and analyzed by flow cytometry. (Scale bar: 50 µm) B) Volcano plot on differential gene expression between two groups (Ctrl and ABCA), *X*‐axis: log2Fold change of expression; *Y*‐axis: ‐log10(FDR). C) Hierarchical clustering of differentially expressed genes between two groups (Ctrl and ABCA), each column represents one sample, and rows represent genes. D) Heat map of different metabolites in Wnt/β‐catenin signaling pathway by ABCA compared to control. E) GSEA result for the wnt/β‐catenin signaling pathway. F,G) The protein expression of β‐catenin, cyclin D1, and C‐myc in LLC cells after drug treatment was measured by Western blot assay. H,I) Colony formation analysis. J) LLC cells were treated with increasing concentrations of drugs, as determined by the MTT assay, after 24 h incubation. K) GSEA result for the Positive Regulation of Cell Cycle Arrest pathway. L,M) Quantitative analysis of Cell cycle profiles of LLC cells induced by the indicated drugs by fluorescence‐activated cell sorting (FACS) analysis. N,O) Quantitative analysis of the apoptosis of LLC cells induced by the indicated drugs, as assessed by FACS analysis. *P* values were determined using a one‐way ANOVA: **, *p *< 0.01; ***, *p *< 0.001.

Considering the critical roles of β‐catenin in both stem cells and normal somatic cells,^[^ ^24^, ^41^ ^]^ it is imperative for ABCA to selectively target and inhibit the Wnt pathway specifically within tumor cells. The cellular uptake analysis in Lewis lung carcinoma (LLC) cells, mouse monocytic macrophage leukemia cell lines (RAW264.7), human bronchial epithelial cells stem cells (Beas‐2B), stem cells from human exfoliated deciduous teeth (Shed), based on findings from confocal laser scanning microscopy and flow cytometry, suggests that ABCA demonstrates enhanced internalization into tumor cells compared to the control and individual AB at an equivalent concentration (**Figure** **4**A; Figure S4A–C, Supporting Information). To investigate the intracellular regulatory effects of ABCA, a comparative analysis of cellular transcriptome sequencing was conducted between cells treated with ABCA and mock‐treated cells. As illustrated in Figure 4B, treatment with ABCA resulted in differential expression of 3007 genes, out of which 1927 exhibited up‐regulated expression while 1080 exhibited down‐regulated expression. This observation was further confirmed by subsequent clustering analysis (*n* = 3) as shown in Figure 4C. More importantly, ABCA‐treated induced significant inhibition in the wnt/β‐catenin signaling pathway (Figure 4D). In comparison to the control cell group, the Gene Set Enrichment Analysis (GSEA) revealed a significant enrichment of suppression signatures in the Wnt/β‐catenin signaling pathway, suggesting negative regulation of both the CA's direct binding target β‐catenin and related Wnt cascades (Figure 4E). Additionally, the western blot analysis confirmed the downregulation of β‐catenin, as well as the decreased expression levels of related C‐myc and Cyclin D1 in the ABCA‐treated cell group (Figure 4F,G). This further elucidates the functional mechanism of ABCA in LLC cells by providing support for its role in suppressing the Wnt/β‐catenin signaling pathway.

The excessive activation of the Wnt pathway has been extensively demonstrated to be strongly associated with the proliferation and metastasis of tumor cells.^[^ ^41^, ^42^, ^43^ ^]^ Motivated by the notable inhibitory effects of ABCA on the Wnt pathway, we proceeded to investigate the antitumor properties of ABCA at the cellular level. As anticipated, ABCA demonstrates anti‐tumor efficacy by impeding cell proliferation and facilitating apoptosis. Based on the results of the colony formation assay presented in Figure 4H,I, it is evident that treatment with ABCA significantly decreased colony formation compared to control, CA, and AB treatments. As shown in Figure 4J, ABCA or CA demonstrated a pronounced intracellular inhibitory effect. This finding was supported by the observation of cell cycle arrest in the group treated with ABCA, as evidenced by a decrease in the proportion of cells in the S‐phase and a significant increase in cells arrested at the G2/M‐phase, as determined through cell cycle analysis (Figure 4K–M). Further examination of the transcriptome sequencing data revealed that ABCA had a notable impact on signaling pathways associated with cellular apoptosis and the cell cycle (Figure 4K). In accordance with these findings, flow cytometry analysis revealed a significantly higher rate of apoptosis in cells treated with ABCA compared to the control group, indicating a pronounced promotion of cellular apoptosis (Figure 4N,O). Overall, these findings collectively suggest that ABCA exhibits anticipated inhibition of Wnt/β‐catenin signaling and synergistic antitumor efficacy in vitro. In conclusion, ABCA can downregulate the expression of β‐catenin protein and significantly inhibit the activation of the Wnt pathway, thereby inducing apoptosis in tumor cells.

### 2.5 The ABCA Demonstrated a Synergistic Enhancement Effect on Anti‐PD1 Immunotherapy in a Murine Model of Lung Adenocarcinoma Orthotopic Allograft

**Figure 5:**
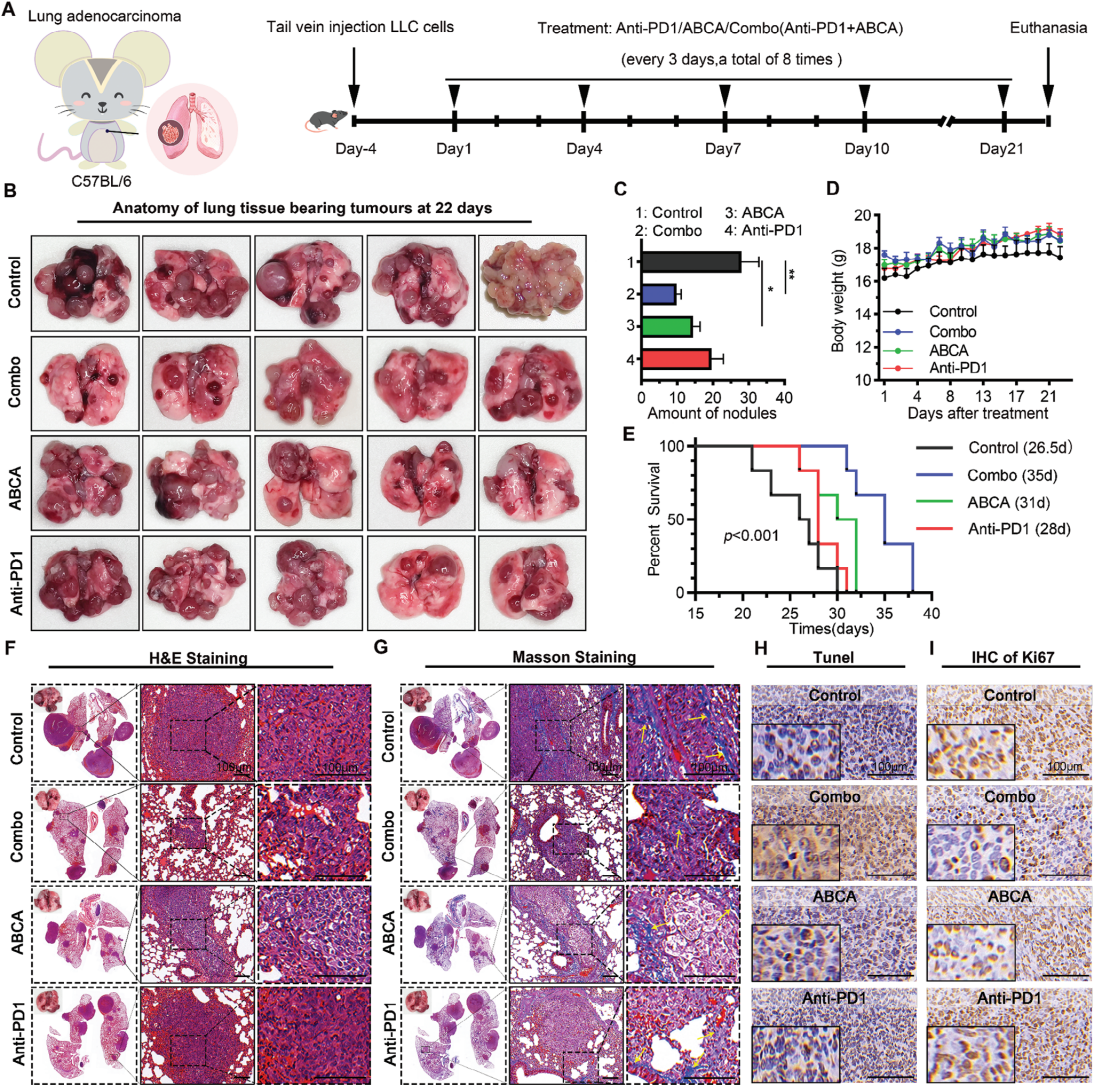
ABCA synergistic enhances immunotherapy in LUAD orthotopic allograft mice model. A) A diagrammatic sketch of the lung adenocarcinoma orthotopic allograft model was established in C57BL/6 mice. B) Photograph of lung tissue of tumor‐bearing from mice treated with PBS, ABCA (3 mg k^−1^g), Anti‐PD1 (3 mg k^−1^g)) and Combo, respectively (*n* = 5 per group). C) Correspondence statistical analysis of a number of the lung tissue nodules with indicated treatment at 22 days (*n* = 5 per group). *P* values were calculated using a one‐way ANOVA: *, *p *< 0.05; **, *p *< 0.01. D) Changes in body weight during the 3 weeks treated with indicated drugs (*n* = 5 per group). E) Survival of mice treated with PBS, Anti‐PD1, ABCA, Combo intravenous injection in LUAD orthotopic allograft mice model. P values were counted by the Log‐rank test (*n* = 6 per group). F,G) The H&E F) and Masson G) staining of pathological sections from lung tissue‐bearing tumors (Scale bar: 100 µm). H)The TUNEL staining of lung tissue‐bearing tumor after indicated treatments (Scale bar: 100 µm). I) IHC staining of Ki67 for lung tumors after indicated treatments for 22 days (Scale bar: 100 µm).

The impressive in vitro findings prompted us to investigate the in vivo effect of ABCA using the lung adenocarcinoma orthotopic allograft model, which was established by intravenously injecting LLC cells. The mice were administered with different substances four days after LLC injection. The experimental groups in this study included PBS (Control group), Anti‐PD1 (PD‐1, 3 mg k^−1^g), ABCA (3 mg k^−1^g), and Combo (ABCA combined with Anti‐PD1, both at a dose of 3 mg k^−1^g), as illustrated in **Figure** **5**A. These administrations were given intravenously every three days for a total of eight times, and the mice were euthanized after 21 days. The lungs bearing tumors, surgically isolated at the end of administration, were fully visualized in Figure 5B. The number of lung nodules did not show a statistically significant difference between the mice treated with anti‐PD1 and the control group (Figure 5C). The number of lung nodules in mice treated with ABCA was observed to be lower compared to those treated with Anti‐PD1, while the combination treatment group exhibited the fewest lung nodules among all treatment groups. These findings suggest that ABCA possesses inherent antitumor properties and synergizes with the immunotherapeutic agent. The survival curves further supported this trend, demonstrating that Anti‐PD1 monotherapy failed to achieve the desired extension of survival (with a median survival time of 28 days). However, when combined with ABCA treatment, Anti‐PD1 therapy significantly prolonged survival without causing abnormal weight loss (with a median survival time of 35 days) (Figure 5D,E). Importantly, these interventions did not result in haematotoxicity or visceral damage, particularly concerning liver and kidney function (Figure S5, Supporting Information).

Furthermore, the immunosensitizing potential of ABCA was confirmed through the application of histopathological and immunohistochemical staining techniques. Specifically, H&E staining and Masson staining results revealed significant improvement in pulmonary fibrosis remission (yellow arrows highlight collagen fibers) and reduction in lesion area in the combination treatment group of mice, indicating an effective inhibition of tumor growth (Figure 5F,G). Additionally, TUNEL staining demonstrated that the ABCA combined with the Anti‐PD1 group exhibited a much more noticeable induction of tumor cell apoptosis compared to the other three groups (Figure 5H). The analysis of tumor cell proliferation using Ki67 immunohistochemical staining also supported these findings by showing the most pronounced down‐regulation in ABCA combined with Anti‐PD1 treatment among all groups (Figure 5I). Collectively, these results demonstrate that the combination of ABCA and Anti‐PD1 effectively suppresses the growth of lung adenocarcinoma orthotopic allograft in vivo, providing an accurate and streamlined approach. Notably, this combined treatment exhibits superior antitumor efficacy compared to monotherapy with PD1 antibody.

### 2.6 The Efficacy of Anti‐PD1 Immunotherapy is Enhanced by ABCA through the Inhibition of the Wnt Signaling Pathway and Augmentation of the Immune Microenvironment

**Figure 6:**
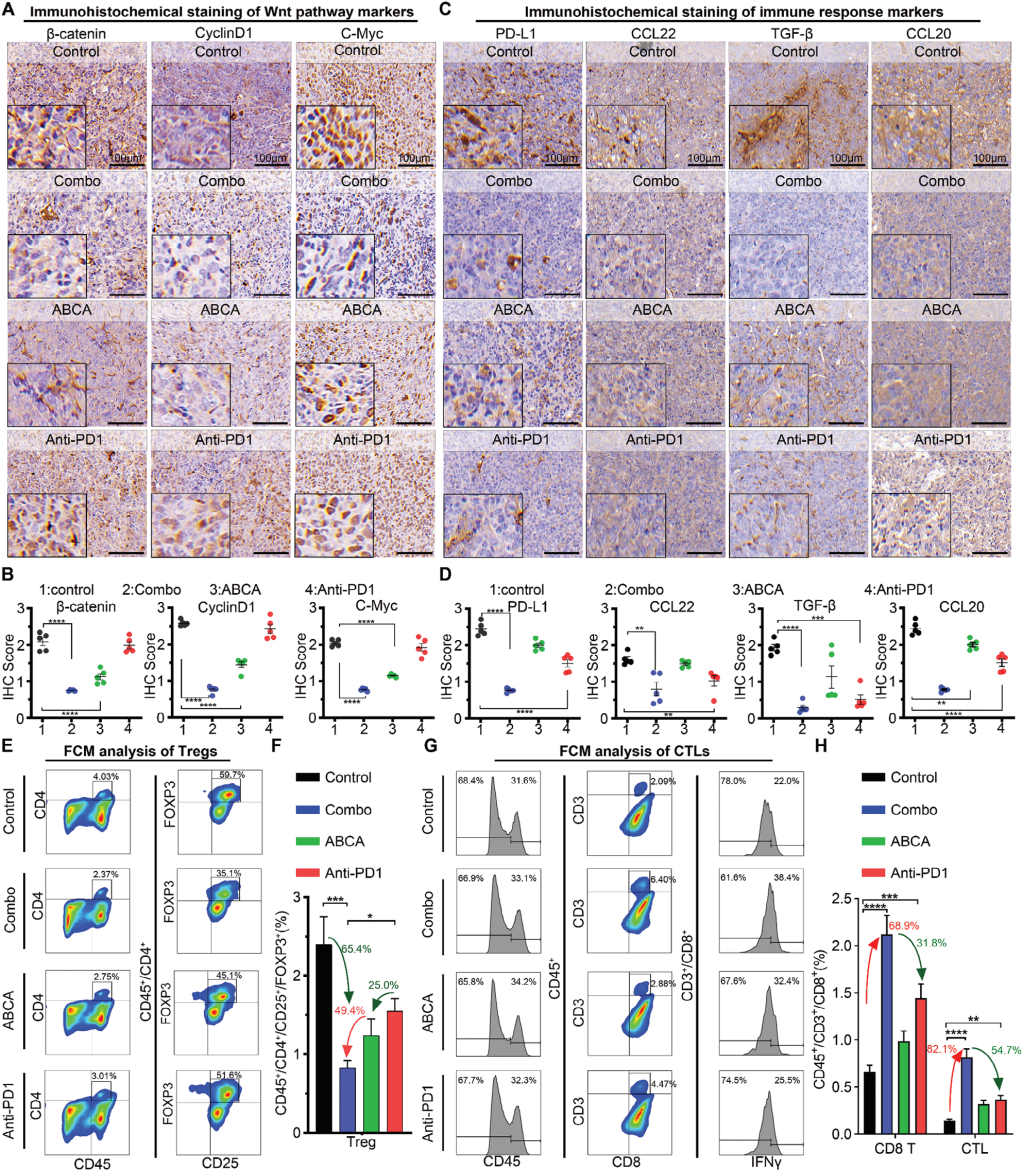
ABCA synergistic enhances immunotherapy in LUAD orthotopic allograft mice model. A) Representative images of IHC staining including β‐catenin, CyclinD1, and C‐myc for lung tumors after indicated treatments for 22 days (Scale bar: 100 µm). B)The quantify scores of β‐catenin, CyclinD1, and C‐myc were performed in dissected lung tumor tissues. *P* values were calculated using a one‐way ANOVA: ***, *p *< 0.001; ****, *p *< 0.0001. C) Representative images of IHC staining including PD‐L1, CCL22, TGF‐β, and CCL20 for lung tumors after indicated treatments for 22 days (Scale bar: 100 µm). D)The quantify scores of PD‐L1, CCL22, TGF‐β, and CCL20 were performed in dissected lung tumor tissues. *P* values were calculated using a one‐way ANOVA: **, *p *< 0.01; ****, *p *< 0.0001. E) Multi‐channel flow cytometry graphs showing the proportions of regulatory T cells (Tregs) E) in lung tumor with different treatments. F) Quantification analysis of the proportion of CD25^+^/Foxp3^+^ cells in lung tumors (*n* = 3, mean ± SD). P values were calculated using a one‐way ANOVA: *,*p *< 0.05; **, *p* < 0.01. G) Multi‐channel flow cytometry graphs showing the proportions of cytotoxic lymphocytes (CTLs) G) in lung tumor with indicated treatments. H) Quantification analysis of the proportion of CD8^+^ IFN‐γ^+^ cells in lung tumors (*n* = 3, mean ± SD). *P* values were calculated using a one‐way ANOVA: *,*p *< 0.05; **, *p *< 0.01.

To further elucidate the anti‐tumor mechanism of ABCA combined with anti‐PD1, we performed Immunohistochemistry (IHC) staining to detect the expressions of β‐catenin, CyclinD1, and C‐myc in lung tumor tissue (**Figure** **6**A). In comparison to the Anti‐PD1 group, there was a significant downregulation in the expressions of β‐catenin and its main downstream targets CyclinD1 and C‐myc, indicating an effective suppression of the Wnt/β‐catenin pathway in vivo (Figure 6A,B). Considering the intimate association between activation of the Wnt signaling cascade and the development of an immunosuppressive microenvironment, it is crucial to investigate the expression profiles of immune factors during ABCA sensitization to Anti‐PD1 therapy. The downregulation of β‐catenin has been reported to effectively suppress the downstream expression of PD‐L1 in the Wnt pathway, thereby facilitating the reactivation of immunotherapy.^[^ ^43^, ^44^ ^]^ The immunohistochemistry results demonstrated a significant decrease in PD‐L1 expression in the ABCA, Anti‐PD1, and combination treatment groups compared to the control group. Notably, the combination therapy group exhibited the most pronounced reduction, indicating a potential modulation of immune evasion signals and augmentation of immune response (Figure 6C,D). The different treatment groups showed disparities in the expression of several immunosuppressive markers or cytokines simultaneously (Figure 6C,D). Specifically, C–C Motif Chemokine 22 (CCL22), a crucial immunosuppressive chemokine involved in the recruitment of regulatory T cells to the tumor microenvironment,^[^ ^45^ ^]^ was found to be decreased in tumor tissues of the combination therapy group. This reduction may be attributed to the interference of CA with β‐catenin/BCL9 interactions,^[^ ^28^ ^]^ leading to a diminished infiltration of immunosuppressive T cells into the tumor and consequently enhancing the responsiveness of ABCA‐treated tumors to Anti‐PD1 therapy. Furthermore, a notable association was observed between heightened levels of TGF‐β expression and diminished effectiveness of PD1/PD‐L1 blockade. Our findings suggest that the co‐administration of ABCA and Anti‐PD1 leads to a reduction in TGF‐β levels in tumor tissues compared to treatment with Anti‐PD1 alone, indicating a potential decrease of the immunosuppressive microenvironment with the combined therapy. Additionally, in the combined group of ABCA and Anti‐PD1, we observed a significant decrease in the intertumoral distribution of C–C Motif chemokine 22 (CCL22), a secretory factor produced by tumor‐associated macrophages that facilitates infiltration of regulatory T cells.^[^ ^46^ ^]^ As anticipated, the multichannel FCM analysis of Tregs revealed a remarkable reduction exceeding 60% in the Treg population within lung tumors following combination treatment with ABCA and Anti‐PD1 (Combo group), compared to treatment with Anti‐PD1 alone (Figure 6E,F). Moreover, the combination therapy of ABCA and Anti‐PD1 (Combo group) exhibited a statistically significant increase of over 30% in CD8 T cells and IFN‐γ expression, surpassing treatment with Anti‐PD1 alone (Figure 6G,H). Collectively, these data suggested that ABCA enhances the efficacy of Anti‐PD1 immunotherapy by modulating the tumor microenvironment through downregulation of immunosuppressive factors and reduction in infiltration of immunosuppressive cells such as Tregs in lung adenocarcinoma.

## 3 Discussion

Non‐small cell lung cancer, particularly lung adenocarcinoma, has become a significant contributor to global cancer‐related mortality due to its high prevalence, aggressive progression, and unfavorable prognosis.^[^ ^47^, ^48^ ^]^ PD1/PD‐L1 blockade has become a primary treatment for NSCLC, but its effectiveness depends not only on PD1/PD‐L1 expression levels but also on the activation of alternative pathways such as the Wnt pathway leading to diminished immune response rates.^[^ ^49^, ^50^, ^51^, ^52^ ^]^ Numerous studies have provided evidence that the classical Wnt/β‐catenin pathway plays a significant role in the development of drug resistance to cancer immunotherapy.^[^ ^53^, ^54^, ^55^ ^]^ The use of Anti‐PD1 antibody alone can lead to accelerated tumor growth, but inhibiting the Wnt/β‐catenin pathway may enhance its efficacy for lung cancer treatment.

The use of combination therapy is a common approach to address treatment resistance and enhance overall efficacy. However, the Wnt/β‐catenin pathway poses challenges for combination with different medications due to off‐target effects. Research has been focused on combining Wnt inhibitors with immunotherapy to optimize therapeutic outcomes.^[^ ^26^, ^27^, ^29^, ^56^, ^57^ ^]^ To date, there have been no clinical successes.^[^ ^58^, ^59^ ^]^ In this study, ABCA was synthesized successfully using a simple and gentle one‐pot method. The resulting ABCA exhibited distinct physicochemical characteristics and a favorable safety profile, as demonstrated by the absence of haematotoxicity, endotoxicity, liver and renal function impairment, and immune‐related adverse reactions in mouse models (Figures 2, 3; Figure S1, Supporting Information). Furthermore, our findings demonstrate that targeting Wnt/β‐catenin signaling with an immunotherapeutic enhancer shows promise as a means of activating the immune response against tumors while preventing resistance to PD1/PD‐L1 blockade and enhancing the effectiveness of immunotherapies.

The potential of ABCA is to make tumors respond better to immunotherapy, which can help overcome resistance to PD1/PD‐L1 blockade. This formulation strategy can also be applied to similar hydrophobic drug small molecules to improve and enhance the therapeutic effect. Similarly, combining Wnt inhibition may also help overcome resistance to chemotherapy and targeted therapies. Our strategy, while showing promise in mouse models, may not fully translate to human patients and will require further experimental clinical validation as well as molecular or formulation optimization. Despite the potential, several challenges need to be addressed before Wnt inhibition can be routinely integrated into cancer treatment, such as timing and dosing, variability in drug metabolism and immune response, pathway complexity, and safety need to be addressed.

## 4 Conclusion

Acquired resistance of immune checkpoint inhibitors (ICBs) has emerged as a significant challenge, posing a negative impact on the generalizability of FDA‐approved immunotherapies such as Anti‐PD‐1. In this study, we present compelling evidence linking the Wnt/β‐catenin signaling pathway to acquired resistance against Anti‐PD1 and propose the development of a novel ICB enhancer. To address the issue of unknown applicability of general immunosensitizers in acquired resistance, we have successfully constructed a co‐assembly ABCA system that combines Wnt suppresser CA and AB through an efficient approach. Utilizing hydrophobic interaction, ABCA demonstrates high loading efficiency while simultaneously suppressing Wnt/β‐catenin signaling and sensitizing PD‐L1 blockade. This dual action eliminates obstacles associated with tumor cell proliferation and restores the tumor immune microenvironment. Through comprehensive in vitro and in vivo experiments, we have demonstrated that ABCA functions as an effective ICB‐enhancer with a favorable biosafety profile and superior anti‐tumor effects compared to Anti‐PD1 monotherapy. In conclusion, our findings offer a promising strategy for developing novel immune checkpoint inhibitor enhancers with potential clinical translation capabilities, providing a new therapeutic tool for highly malignant solid tumors characterized by complex immune microenvironments like LUAD.

## 5 Experimental Section

### General Remarks

Phosphate buffer saline (PBS) was originated from Gibco. HAuCl_4_·xH_2_O, carnosic acid (CA), and Tris (2‐carboxyethyl) phosphine hydrochloride (TECP) were purchased from Aladdin Chemicals. All other reagents were used in this experiment which obtained from Sigma‐Aldrich, not otherwise specified.

### Bioinformatics Analysis of GEO Datasets

The transcriptomic datasets of lung cancer patients obtained from GEO datasets (GSE181820), containing information on response to Anti‐PD1 immunotherapy. The GSE181820 dataset comprised 22 human lung cancer RNASeq samples, including clinical information and related datasets article showed that high TMB group A (*N* = 6) did not response to Anti‐PD1 therapy and low TMB group C (*N* = 6) response to Anti‐PD1 therapy.

### Synthesis of ABCA

As a first step, 500 µL AB protein (in PBS buffer, 10 mg mL^−1^) and 500 µL TECP (in PBS buffer, 1 mg mL^−1^) were mixed and reacted for 10 min using an ultrasound instrument. Subsequently, PBS solution (3.5 mL) was slowly added into the solution described above. Next, CA (2 mg dissolved in 4 µL DMSO) was slowly added dropwise into the AB and TECP mixture solution, which was reacted for 20 min in the ultrasonic device. The last step, an aqueous solution of chloroauric acid solution (HAuCl_4_·xH_2_O, 10 mm, 500 µL) was added to the mixture for 10–20 min in the sonicator, obtaining the yellowish solution, named ABCA.

### Physicochemical Properties of ABCA

The particles size distribution and zeta potential of the ABCA solution were measured by dynamic light scattering (DLS) (Malvern‐Zetasizer Nano ZS system). The surface of the chemical structure of ABCA was evaluated by Micro‐Infrared Spectroscopy (Bruker VERTEX70) and UV spectroscopy. The surface structure of morphology and lattice was imaged by Lorenz Transmission Electron Microscope (Talos F200X) and 120KV TEM instrument (Talos L120C G2).

### Cellular Uptake

The LLC, Shed, Raw264.7, and Beas‐2B cells were uniformly spread in 35 mm confocal dishes and six‐well plates, at a density of 5 × 10^4 ^cells mL^−1^ in each confocal dish and 1 × 10^5^ cells mL^−1^ in each well of the six‐well plate, with a blank control group, an AB‐administered group, and an ABCA‐administered group. After the cells were adhered to the wall overnight, 20 µg mL^−1^ of FITC fluorescence‐labeled AB and ABCA was added to the dishes and the six‐well plates, respectively, and incubated for 6 h. Cells were processed in confocal dishes: first discarding the cell supernatant in the dishes, washing twice with PBS, and adding paraformaldehyde to fix the cells for 10 min, then staining the cell nuclei with DAPI staining solution, and finally taking pictures with a confocal laser scanning microscope. Cells were processed in six‐well plates: discarding the cell supernatant, washing it twice with PBS, digesting the cells with trypsin, then centrifuged to collect the cell precipitate, and the cells were resuspended in 300 µL of PBS to detect by flow cytometry.

### Transcriptome Sequencing

Preparing six 10 cm cell culture dishes, 5 × 10^5^ LLC cells were evenly distributed each dish, which were divided into a blank control group and a dosing group, with three samples in each group. After the cells were attached to the wall overnight, 12 µg mL^−1^ of ABCA was added to each dish in the drug administration group, and an equal amount of PBS was added to each dish in the blank control group, and then incubated for 24 h. The supernatant of the cells was discarded and washed with PBS twice, then 1 mL TRIzol reagent was added to each dish, and cells were collected into RNAase‐free EP tubes for transcriptome sequencing.

### Western Blot

The drug‐treated cell precipitate was collected into EP tubes and lysed with configured RIPA lysate and PMSF (100:1) and then centrifuged at 12 000 rpm for 20 min, and subsequently, the supernatant was taken to prepare for Western blot samples, along with the concentration of protein samples was detected using the BCA Protein Quantification Kit (Biyuntian, China). The above protein samples were subjected to SDS‐PAGE electrophoresis, and the electrophoresis time was judged according to the protein maker. After electrophoresis, the PVDF membrane was used for protein blotting, followed by blocking the membrane with 5% skimmed milk, generally for 2 h. The membrane was then incubated with primary antibody overnight at 4 °C, then with secondary antibody for 1 h, and finally, chemiluminescence was performed.

### Clone Formation Assay

LLC cells (1 × 10^3^) were evenly spread in 12‐well plates, and after adhering to the wall overnight, drugs were added to treat the cells, which were replaced with fresh complete medium after 24 h, and then continued to cultivate the cells for 7 days. When the cells were observed the obvious clusters of cells in the well plates, the supernatant was discarded, and the cells were fixed with paraformaldehyde and then stained with crystal violet staining solution, and finally photographed to count the number of clones of the cells in each group.

### CCK‐8 Assay

Cells (3 × 10^3^) were evenly spread in 96‐well plates, overnight, and then the cells were treated with drugs at a maximum concentration of 200 µg mL^−1^, sequentially diluted 2 times to establish 10 concentration gradients. After adding drugs for 24 h, the cell proliferation detection reagent CCK‐8 was added, then the OD value at 450 nm was detected by Microplate Reader, and finally, the data were statistically analyzed.

### Flow Cytometry to Detect Cell Cycle

Cells (3 × 10^4^) were evenly spread in a 6‐well plate, and after being adhered to the wall overnight, the cells were treated with drugs and collected after 24 h. The cells were fixed with 70% ethanol at −20 °C overnight. Cells were stained with 0.4 mL PI/RNase Staining Buffer (BD, Cat. No. 550 825) for 15 min at room temperature and analyzed by flow cytometry.

### Flow Cytometry to Detect Cell Apoptosis

Cells were collected using the previous method, then resuspended by 250 µL of 1 × Binding Buffer, followed by the addition of 5 µL Annexin V/PE (BD, Cat. No. 559 763) and 5 µL 7‐AAD (BD, Cat. No. 559 763), mixed and analyzed by flow cytometry.

### Hemolysis Test

Whole blood was collected from the eyeballs of C57BL/6 mice into an anticoagulant tube, followed by centrifugation at 1500 rpm for 10 min. The erythrocytes were then washed repeatedly with 0.9% saline 3–4 times until the supernatant became nearly colorless. Subsequently, 2 mL of erythrocytes were diluted with 0.9% saline to prepare a 2% erythrocyte suspension. The 2% erythrocyte suspension was then mixed with ABCA at concentrations of 3, 10, 20, and 40 mg k^−1^g. Then, 0.9% saline served as the negative control (0% hemolysis), while distilled water was used as the positive control to represent 100% hemolysis. The various concentrations of ABCA and a 2% erythrocyte suspension were combined and incubated at 37 °C for 3 h. Subsequently, the mixture was centrifuged at a low speed for 10 min. The supernatant was then transferred to a 96‐well plate, and the absorbance was measured at 545 nm. The rate of hemolysis was calculated using the following formula:^[^ ^60^ ^]^

(1) $$\mathrm{Hemolytic}\mathrm{percentage}\left(\%\right)=\left(At-An\right)/\left(Ap-An\right)\times 100\%$$

where At is the absorbance value of the experimental sample, An is the negative control and Ap is the positive control, respectively.

### Animal Ethics

All experimental animals were purchased from the Laboratory Animal Center (LAC) of Xi'an Jiaotong University. All animal experimental research strictly adheres to Institution Guidelines and approved by the Laboratory Animal Center of Xi'an Jiaotong University (No. 2021‐1734).

### In Vivo Safety Assessment

The healthy C57BL/6 mice (4–5 weeks old, female) were randomly divided into each group (*n* = 6). Then, the PBS group, ABCA group (10 mg k^−1^g), ABCA group (20 mg k^−1^g) and ABCA group (40 mg k^−1^g) were intraperitoneally administrated once every other day for 12 days, respectively. After the last administration, all mice were euthanized. Among them, the whole blood was detected by blood routine examination, and the serum was used for the function of the liver and kidney. Main organs and tissues were immediately fixed with 4% formalin solution and then paraffin‐embedded tissues for H&E staining. The expression of inflammatory factors (IFN‐γ, TNF‐α, IL‐10, IL‐6, and IL‐2) in organs including the heart, liver, kidney, and serum was operated according to the Biolegend protocol (LEGENDplex) and detected using a Flow cytometer (BD Biosciences) instrument.

### In Vivo Biodistribution Analysis

The biodistribution of ABCA was conducted by subcutaneously injecting LLC cells (1 × 10^6^ cells per mouse) into 5–6 week‐old C57BL/6 mice. Upon reaching an approximate tumor volume of 500 mm^3^, 200 µL of ABCA (3 mg k^−1^g) was administered intravenously at time points 0, 4, 6, 18, 24, 48, 120, and 168 h. The distribution of gold (Au) within various organs and tumors was subsequently quantified using inductively coupled plasma mass spectrometry (ICP‐MS).

### LUAD Orthotopic Allograft Mice Model

4–5 weeks old female mice were intravenously injected with LLC cells (8 × 10^5 ^cells per mouse), and then the physical condition of the mice was observed for three days. According to the body weight, all mice were randomly divided into four groups and respectively injected intravenously PBS, ABCA (3 mg k^−1^g), Anti‐PD1(3 mg k^−1^g), and ABCA(3 mg k^−1^g) combined with Anti‐PD1(3 mg k^−1^g) on the fourth day, once every three days lasting 8 times. During the administration period, mice body weights were measured using electronic scales. Besides, the survival curve of mice (*n* = 6 per group) was evaluated in the LUAD orthotopic allograft mice model after the end of the fifth administration. Mice body weight and survival state were recorded on a regular daily schedule, and then the survival curve was monitored the status of the mice until they confirmed to be completely dead. In addition, the lung tumor‐bearing tissue was performed using H&E staining. It was worthwhile to state that the primary antibodies used in immunohistochemical were as follows: anti‐Ki‐67 (CST, USA; 1:400), anti‐β‐catenin (Abcam, USA; 1:100), anti‐CyclinD1(CST, USA; 1:200),Anti‐C‐Myc (Abcam, USA; 1:200),Anti‐PD‐L1(Proteintech, USA; 1:5000),Anti‐TGF‐β (Abcam, USA; 1:400), Anti‐CCL22 (Abcam, USA; 1:100) and Anti‐CCL20 (Abcam, USA; 1:400).

### Statistical Analysis

All raw data were collected from at least three independent samples unless otherwise indicated. Data analyses were performed using a two‐sided Student's *t*‐test for the comparation between the two groups. ANOVA test was used for the comparison of more than two groups. *P *< 0.05 was considered statistically significant. Data were expressed as mean ± s.d. or s.e.

## Conflict of Interest

The authors declare no conflict of interest.

## Supporting information



Supporting Information

## Data Availability

The data that support the findings of this study are available from the corresponding author upon reasonable request.
