# Supplementary material for: Rebooting the Adaptive Immune Response in Immunotherapy‐Resistant Lung Adenocarcinoma Using a Supramolecular Albumin
Source: Small. 2024 Oct 21;20(52):2404892. doi: 10.1002/smll.202404892 (PMC11673449; doi:10.1002/smll.202404892)
Supplement: Supplementary file 1 — Supporting Information [file SMLL-20-2404892-s001.docx]

Supporting Information

**Rebooting the adaptive immune response in immunotherapy-resistant lung adenocarcinoma using a supramolecular albumin**

*Fanni Li^1,^, Jingmei Wang^2^, Tianya Liu^2^, Wenguang Yang^1^, Yong Li^3,4^, Jin Yan^1,3,4*^, Wangxiao He^1,2*^*

F. Li, W.Yang

Department of Medical Oncology and Department of Talent Highland, The First Affiliated Hospital of Xi’an Jiaotong University, Xi’an 710061, PR. China.

J. Wang, T. Liu

Institute for Stem Cell & Regenerative Medicine, The Second Affiliated Hospital of Xi’an Jiaotong University, Xi’an 710004, China

Y.Li

Department of infectious Diseases and Department of Tumor and Immunology in precision medical institute, The Second Affiliated Hospital of Xi'an Jiaotong University, National & Local Joint Engineering Research Center of Biodiagnosis and Biotherapy, The Second Affiliated Hospital of Xi'an Jiaotong University, Xi'an, 710004, PR. China.

J. Yan

Department of Medical Oncology and Department of Talent Highland, The First Affiliated Hospital of Xi’an Jiaotong University, Department of infectious Diseases and Department of Tumor and Immunology in precision medical institute, The Second Affiliated Hospital of Xi'an Jiaotong University, National & Local Joint Engineering Research Center of Biodiagnosis and Biotherapy, The Second Affiliated Hospital of Xi'an Jiaotong University, Xi'an, 710004, PR. China.

Email: [yanjin19920602@xjtu.edu.cn](mailto:yanjin19920602@xjtu.edu.cn) (J. Yan)

W. He

Department of Medical Oncology and Department of Talent Highland, The First Affiliated Hospital of Xi’an Jiaotong University, Institute for Stem Cell & Regenerative Medicine, The Second Affiliated Hospital of Xi’an Jiaotong University, Xi’an 710004, China

Email: [hewangxiao5366@xjtu.edu.cn](mailto:hewangxiao5366@xjtu.edu.cn) (W. He)

^†^ These authors contributed equally.

^*^ Corresponding authors:

^
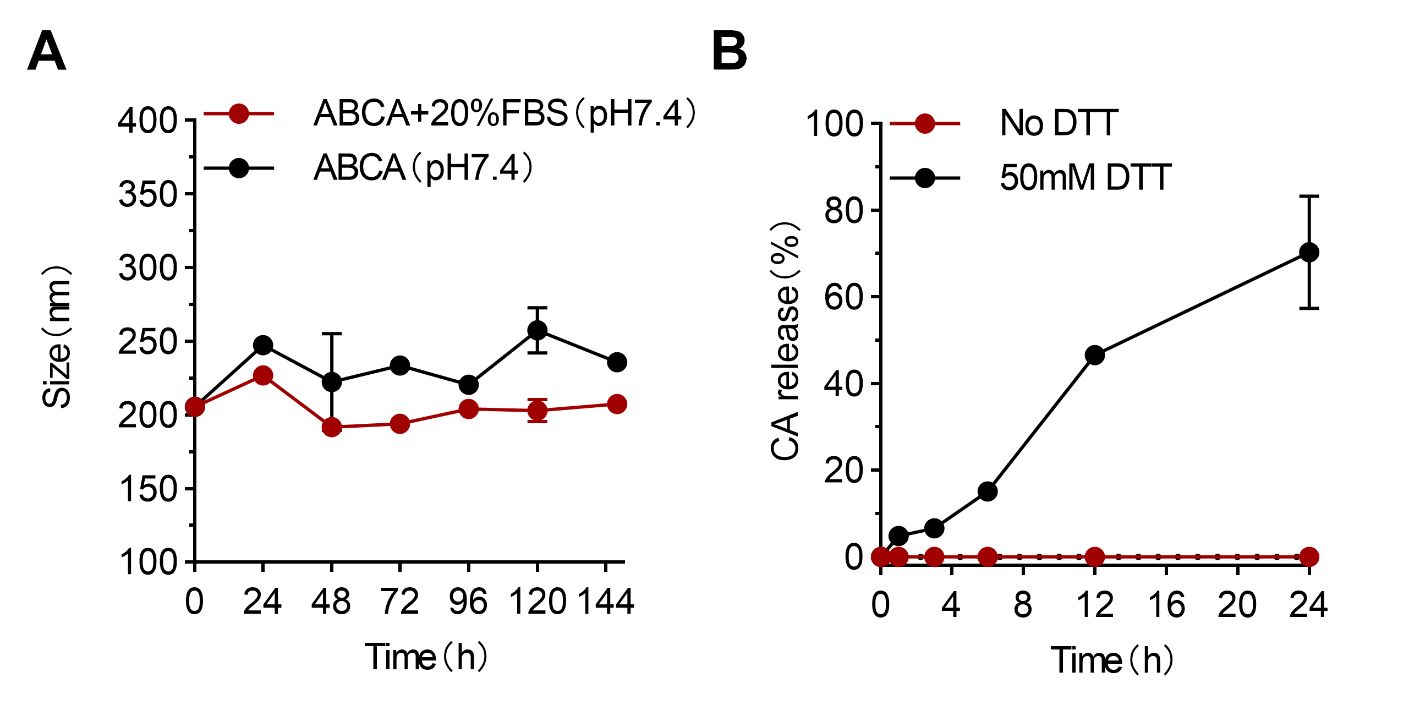
^

**Figure S1**. Synthesis and characterization of ABCA. **A**) Determination of particle size of ABCA in 20% serum at different time points. **B**) High performance liquid chromatography (HPLC) was employed to quantify the release rate of CA from ABCA, under conditions of either no treatment or treatment with 50 mM DTT

^
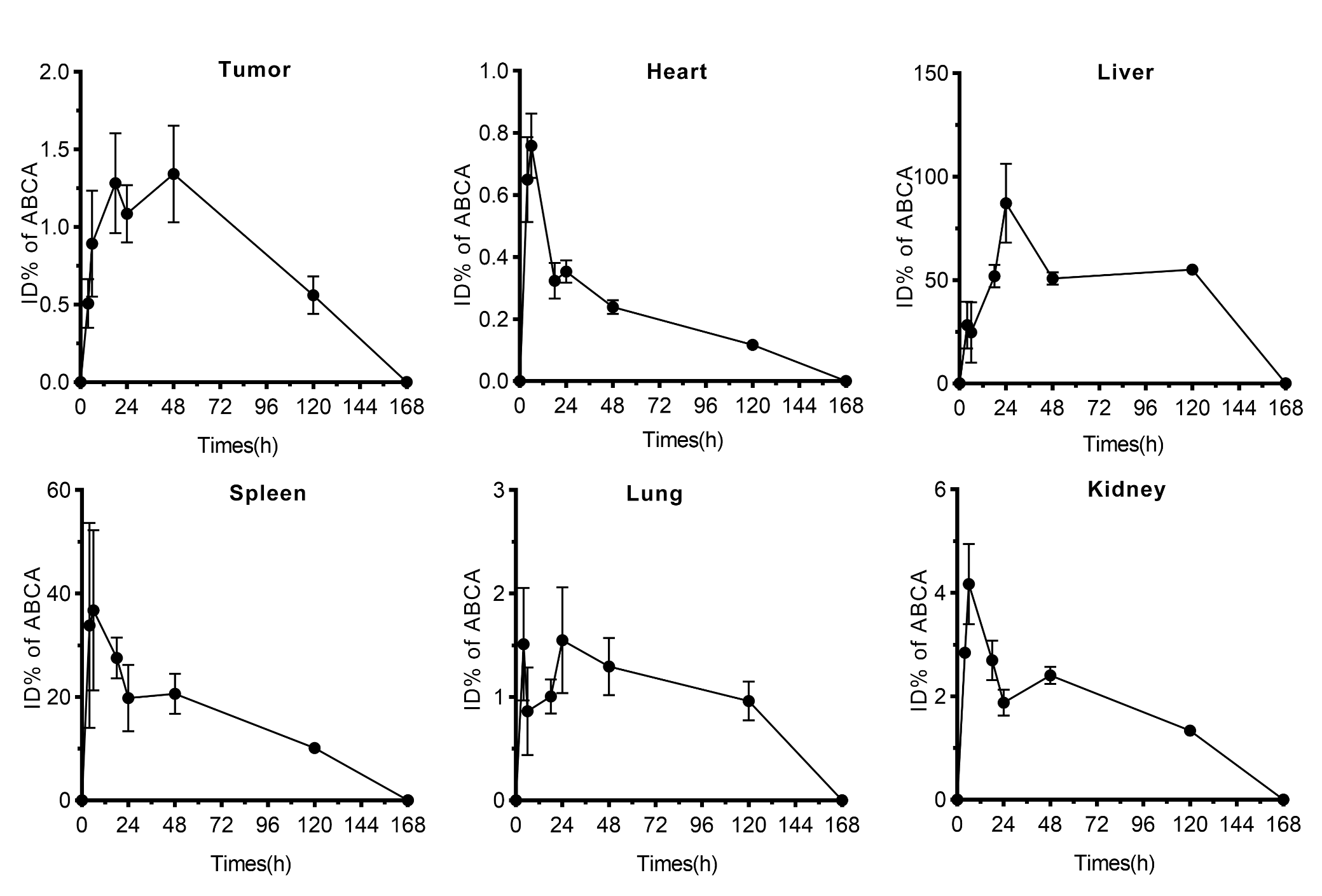
^

**Figure S2.** Biosafety assessment of ABCA in mice. The organ and tumor distribution of gold (Au) were analyzed following the intravenous administration of 3 mg/kg of ABCA in subcutaneous tumor-bearing mice with Lewis lung carcinoma (LLC). Gold concentrations in various tissues, including the heart, liver, spleen, lung, kidney, and tumor, were quantified using inductively coupled plasma mass spectrometry (ICP-MS) at time points of 0, 4, 6, 18, 24, 48, 120, and 168 h post-injection. The data are presented as mean ± standard error (SE).

^
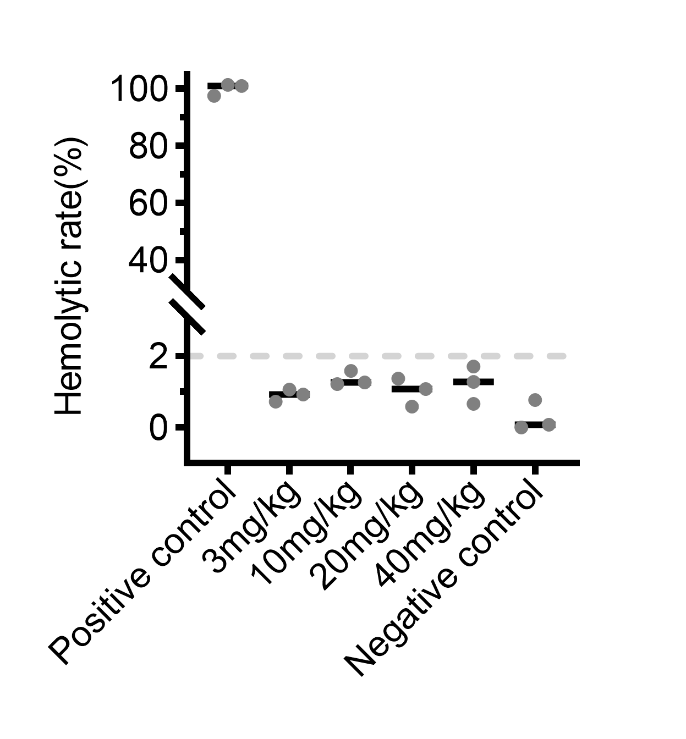
^

**Figure S3.** Biosafety assessment of ABCA in mice. Hemolytic activity assay of ABCA in healthy mice.


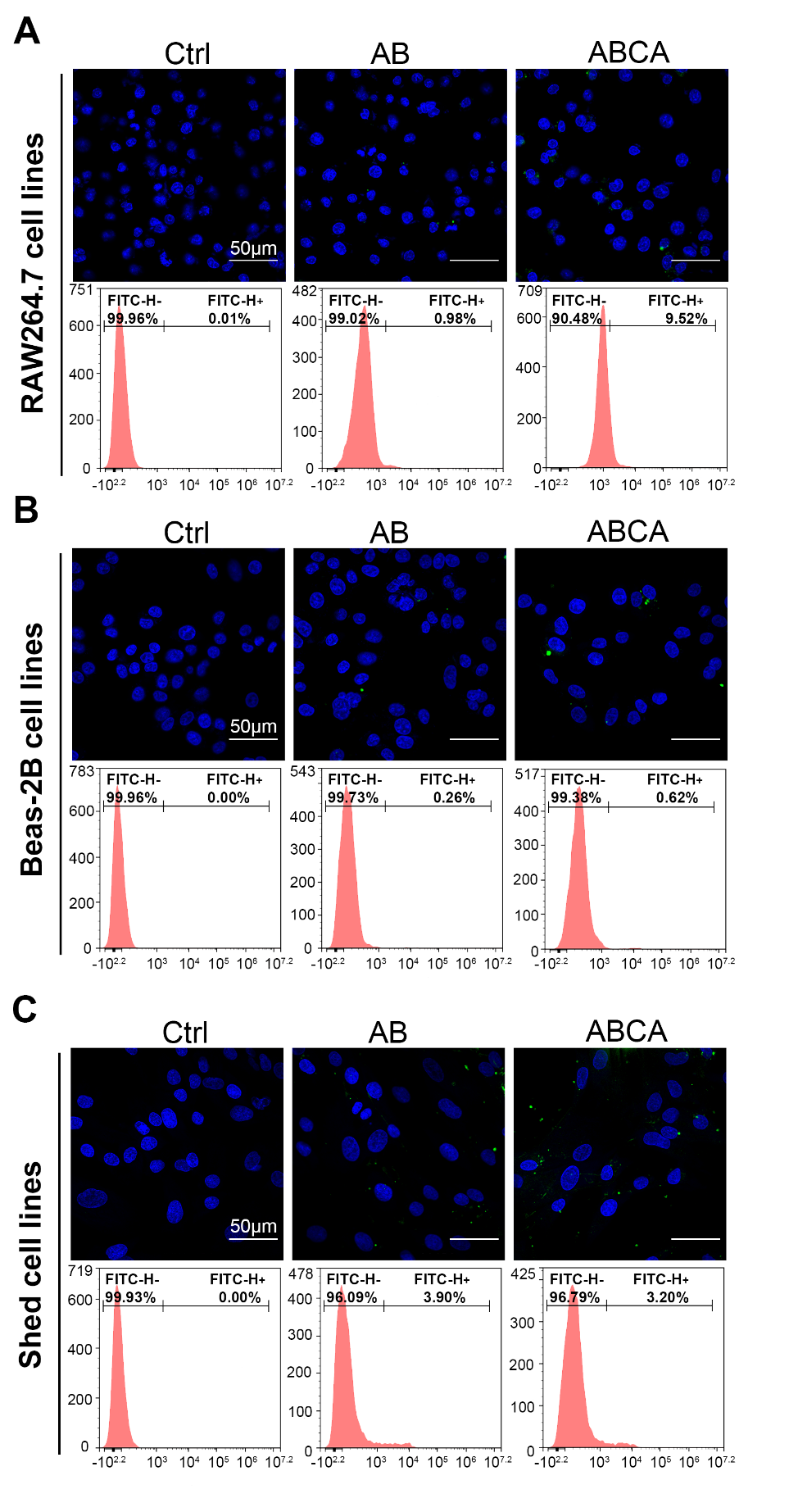


**Figure S4.** *In vitro* anticancer efficacy and mechanism of ABCA on Raw264.7 cells **A**), Beas

-2B cells **B**) and Shed cells **C**) Cellular uptakes of ^FITC^AB and ^FITC^ABCA by LLC cells at 6 h, observed by fluorescence microscope, and analyzed by flow cytometry. (Scale bar: 50 µm).


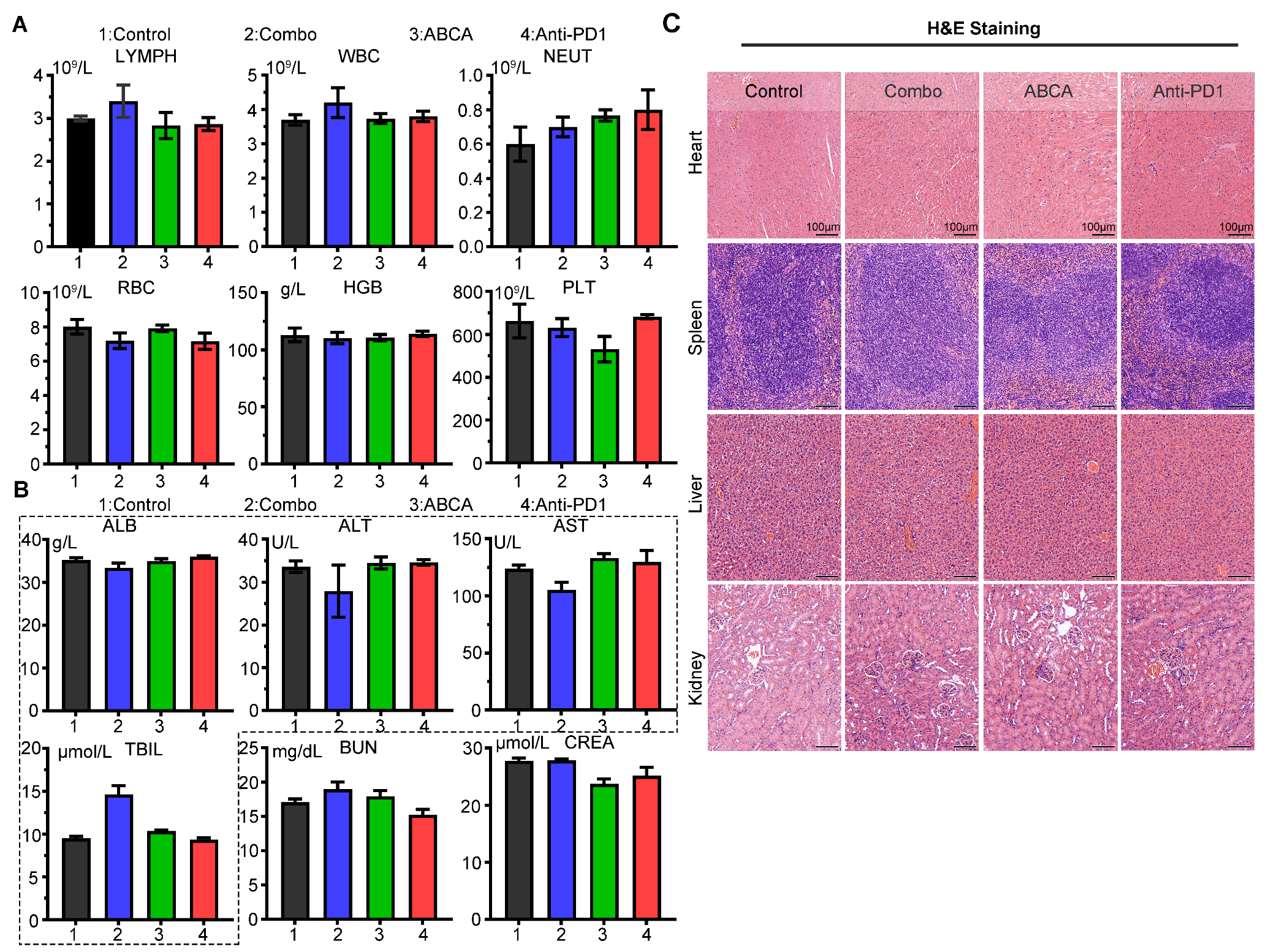


**Figure S5.** **A)** Blood Routine Indexes in mice LUAD orthotopic allograft model with the indicated treatments, respectively. Lymphocytes (LYMPH), white blood cells (WBC), neutrophils (NEUT), Red blood cells (RBC), hemoglobin (HGB), platelets (PLT). **B**) Hepatic and Renal function in mice LUAD orthotopic allograft model with the indicated treatments after 22 days. Albumin (ALB), alanine transaminase (ALT), aspartate aminotransferase (AST), and total bilirubin (TBIL), Blood Urea Nitrogen (BUN), CREA serum creatinine. **C**) Pathological Images of H&E Staining of Heart, Spleen, Liver and Kidney in mice LUAD orthotopic allograft model with the indicated treatments (scale bar: 100 μm).


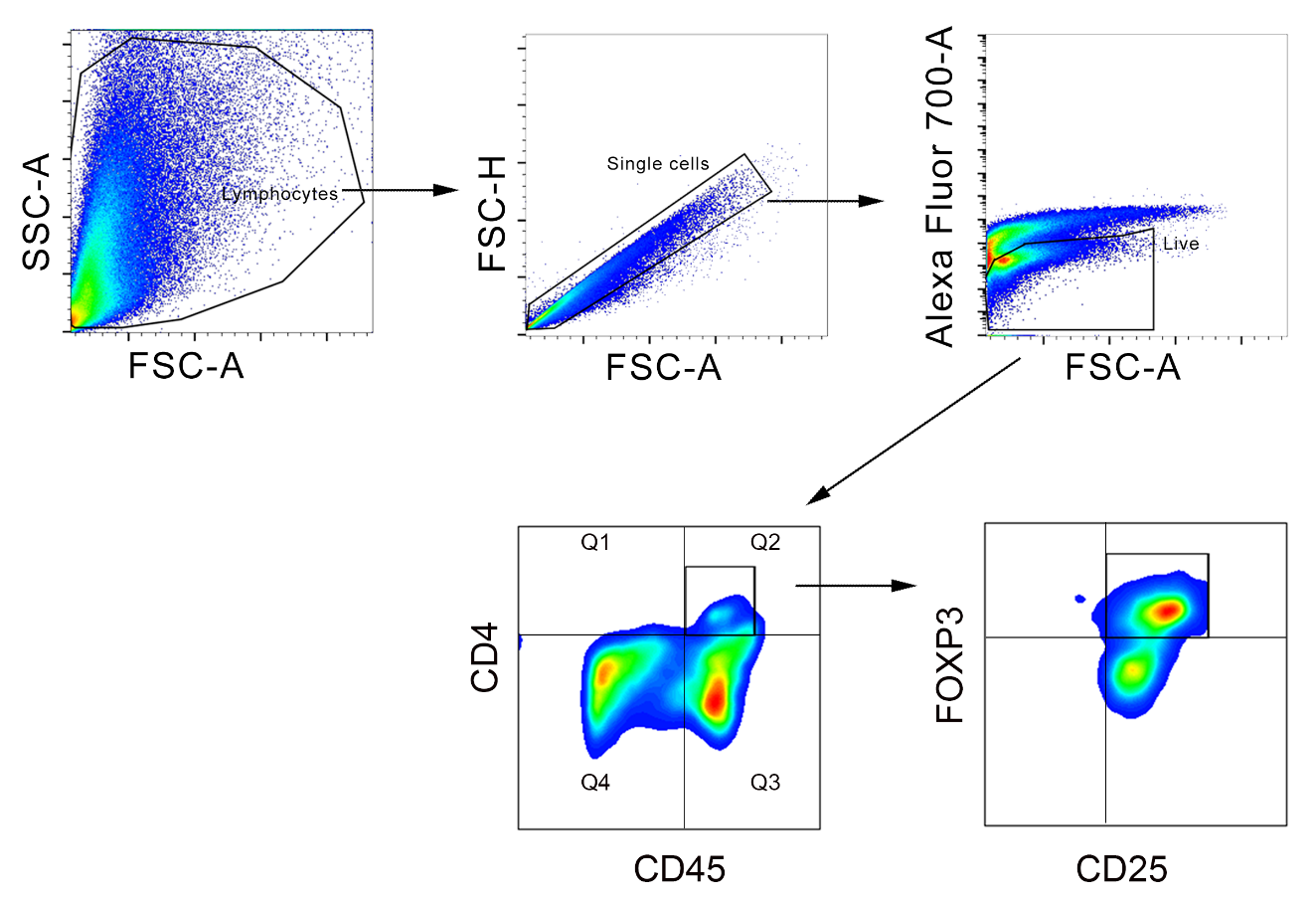


**Figure. S6.** A representative flow cytometry-based gating strategy for identifying Tregs in lung tumors in a mouse model of lung adenocarcinoma. Tregs, regulatory T cells.


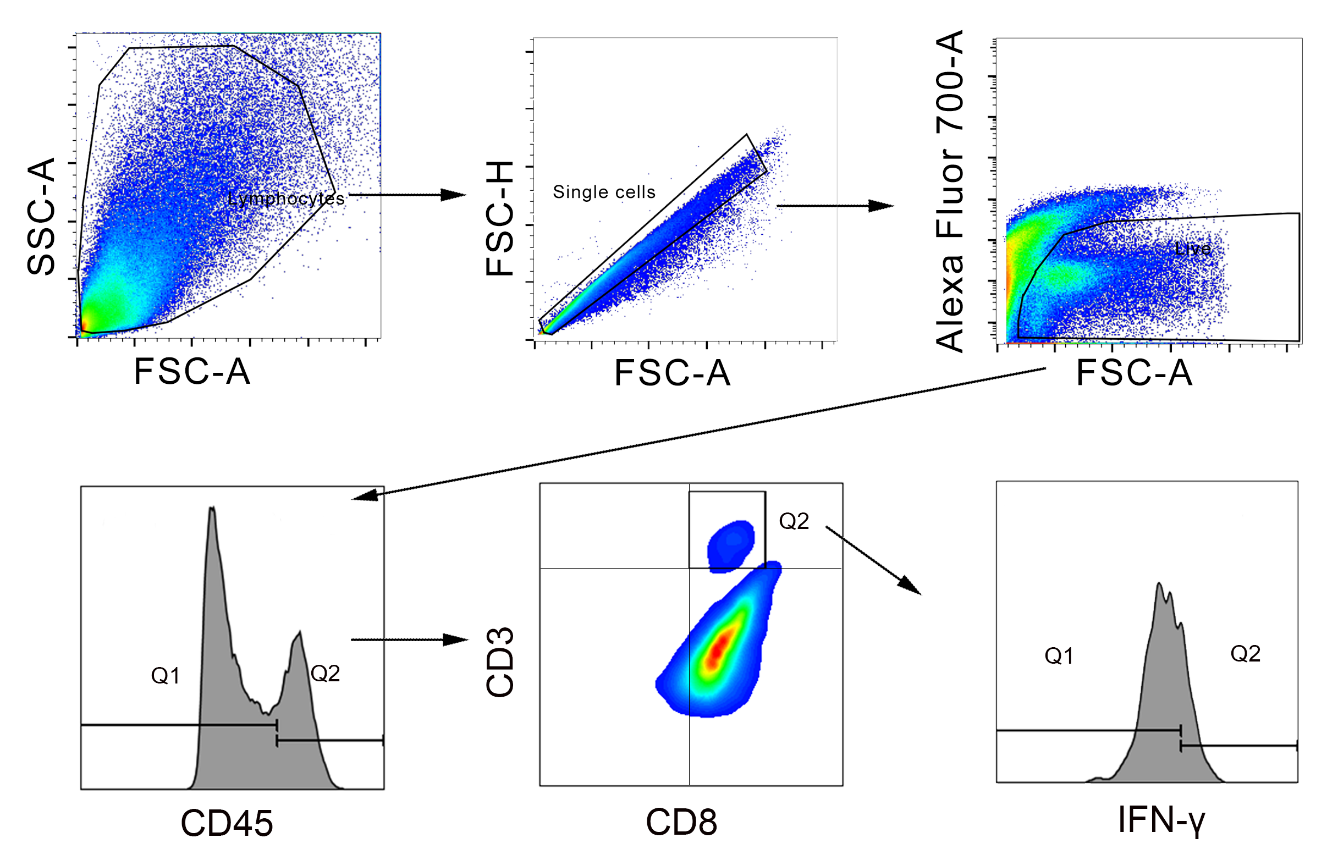


**Figure. S7.** A representative flow cytometry-based gating strategy for identifying CTLs in lung tumors in a mouse model of lung adenocarcinoma. CTLs, cytotoxic lymphocytes.
